# Ketogenic Diet in Obesity and Diabetes: A Narrative Review

**DOI:** 10.3390/nu18122004

**Published:** 2026-06-20

**Authors:** Yousun An, Nicholas Norris, Donglai Li, Jenny E. Gunton

**Affiliations:** 1 Centre for Diabetes, Obesity and Endocrinology, Westmead Institute for Medical Research, University of Sydney, Westmead, NSW 2145, Australia; 2 Faculty of Medicine & Health, University of Sydney, Sydney, NSW 2006, Australia; 3 Department of Diabetes and Endocrinology, Westmead Hospital, Westmead, NSW 2145, Australia

**Keywords:** KD, obesity, diabetes, weight loss, glycaemic control, β-cells, immune response, gut health

## Abstract

A ketogenic diet (KD) is a low-carbohydrate, high-fat dietary approach. Beyond treating neurologic disorders, KDs have attracted significant media attention for their potential to improve obesity and diabetes. The diet induces a metabolic shift from glucose toward fatty acid oxidation and ketone body production. This shift leads to ketosis, which may reduce hunger, partly through the anorexigenic effects of ketone bodies, thereby contributing to weight loss and improved metabolic parameters, including glycaemic control and insulin sensitivity. In particular, the positive effects of KDs lower insulin demand and may thereby improve β-cell function. However, the long-term efficacy, safety, and sustainability of KDs, especially for diabetes, remain debated. This review offers current insights into the effects of ketogenesis and ketosis, as well as the potential mechanisms underlying them. We explore the metabolic effects of KDs in obesity and diabetes, drawing on preclinical and clinical studies, and suggest that combining KDs with antidiabetic agents may provide synergistic benefits. However, combining KDs with these pharmacotherapies, particularly SGLT-2 inhibitors, requires careful clinical supervision because of potential risks, including euglycaemic diabetic ketoacidosis. We explore how a KD alters the composition of the gut microbiota, thereby affecting host health. We conclude by highlighting challenges and future directions for optimising KD-based therapies and by outlining the limitations of the current review.

## 1. Introduction

Intentional dietary planning can have substantial effects for health improvement. Currently, a diverse array of dietary approaches for weight loss and improved metabolism exists, including low-carbohydrate, plant-based, Mediterranean, ketogenic, intermittent fasting, anti-inflammatory, and specialised diets tailored to specific objectives and nutritional profiles. Although the outcomes vary significantly due to individual genetic and lifestyle factors, a clear association between diet, gut health, and metabolic health is evident.

Recent experimental and clinical studies highlight the therapeutic potential of food-supplement strategies for improving overall health outcomes. Ketogenic diets (KDs)—characterised by a high-fat, adequate-protein, and low-carbohydrate composition—have gained considerable attention in recent years as a popular intervention for weight loss and due to their various health benefits [1,2,3]. KDs were used by Frederick M. Allen and Elliot Joslin to prolong life in people with type 1 diabetes (T1D) before the discovery of insulin [4]. They used a diet with up to 70% of calories from fat [4,5]. Russell Wilder introduced KDs in 1921 as a dietary strategy for treating epilepsy [6]. In addition to their established efficacy in managing epilepsy, KDs are associated with beneficial effects in a variety of health conditions, including cancer, neurological disorders, obesity, type 2 diabetes (T2D), and gastrointestinal and respiratory disorders [3,7].

The macronutrient composition of a KD is characterised by a high fat content, usually 55% to 60% of total caloric intake, with carbohydrates restricted to less than 10% and protein accounting for the remainder (30–35%) [3]. The classical ketogenic diet is more restrictive and is typically defined by a 4:1 ratio of fat to the combined amount of protein and carbohydrates, providing approximately 85–90% of total caloric intake from fat [8]. KDs can be categorised into several subtypes based on macronutrient ratios, including the classic long-chain triglyceride (LCT)-based KD, the medium-chain triglyceride (MCT)-based KD, the modified Atkins diet, and the low-glycaemic-index treatment, in which carbohydrates are restricted in amount and are low-glycaemic-index [3,9]. Importantly, not all low-carbohydrate diets induce nutritional ketosis. KDs represent a specific subset of low-carbohydrate diets in which carbohydrate restriction is sufficient to sustain ketone production and nutritional ketosis. KDs represent a heterogeneous group of dietary strategies rather than a single standardised intervention. These differences can significantly influence metabolic outcomes, including body weight, glycaemic control, lipid profiles, inflammation, changes in the gut microbiota and diet adherence [8]. Such variations may substantially influence the degree of ketosis, metabolic adaptation, gut microbiota composition, treatment adherence, and the relative contribution of caloric restriction versus ketosis-specific mechanisms. Therefore, interpretation of KD-related outcomes should account for the heterogeneity of KD protocols.

The current narrative review examines recent mechanistic and clinical evidence on the metabolic and cellular effects of KDs in obesity and diabetes, drawing on clinical trials, animal studies, and key mechanistic investigations identified primarily through PubMed searches. Although formal systematic review methodologies were not applied, given the narrative nature of this review, particular emphasis is placed on KD-induced metabolic adaptations, interactions with the microbiome, and the interplay between KDs and antidiabetic therapies. Together, these factors may contribute to both the therapeutic benefits and potential risks of KDs in metabolic disorders.

## 2. Mechanisms of Action: Effects of Ketogenesis and Ketosis on Cellular Mechanisms

**Figure 1:**
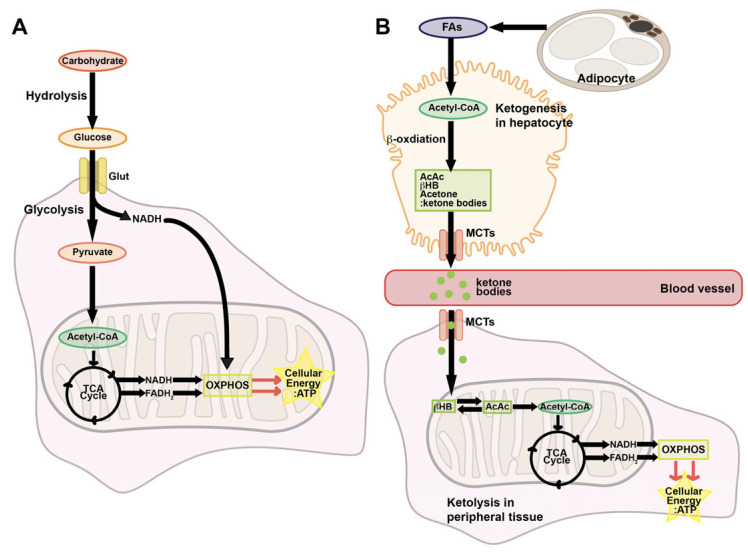
Glucose and ketone body metabolism in relation to carbohydrate availability. (**A**) Under sufficient carbohydrate intake, glucose serves as the primary energy substrate, and ATP is generated via glycolysis, the TCA cycle, and oxidative phosphorylation. (**B**) Under carbohydrate restriction, fatty acids become the main fuel. In the liver, adipocyte-derived fatty acids undergo β-oxidation to produce acetyl-CoA, which is converted into ketone bodies (AcAc, βHB, acetone), released into the circulation and utilised by peripheral tissues as an alternative energy source. Abbreviations: AcAc, acetoacetate; ATP, adenosine triphosphate; βHB, β-hydroxybutyrate; FAs, fatty acids; FADH_2_, Flavin Adenine Dinucleotide; Glut, glucose transporter; MCTs, monocarboxylate transporters; NADH, Nicotinamide Adenine Dinucleotide; OXPHOS, oxidative phosphorylation; TCA cycle, tricarboxylic acid cycle.

Under normal dietary conditions, glucose is the main fuel for cellular energy production via glycolysis, the tricarboxylic acid (TCA) cycle, and mitochondrial oxidative phosphorylation (OXPHOS) (Figure 1A) [10]. However, when carbohydrate intake is restricted, or during prolonged fasting, fatty acids (FAs) released from adipocytes become the predominant energy source [11]. Under these physiological conditions, adipose tissue releases FAs, which are absorbed by the liver and converted into circulating ketone bodies, including acetoacetate (AcAc), β-hydroxybutyrate (βHB), and acetone, which are derived from excess acetyl-CoA produced by fatty acid oxidation [12]. The metabolic effects of KD may also depend on the depth of ketosis achieved, which is commonly assessed based on circulating βHB concentrations. Nutritional ketosis is generally characterised by blood βHB levels of approximately 0.5–3.0 mmol/L [11]. The aim of KDs is to induce a metabolic state known as nutritional ketosis while avoiding pathological ketoacidosis. It should be noted that not all studies included in this review confirmed nutritional ketosis, as ketone bodies were not always measured. The observed metabolic outcomes described here may reflect both dietary carbohydrate restriction and variable degrees of ketosis. Therefore, a limitation of this review is that not all studies implementing KDs confirmed or quantified nutritional ketosis.

The liver is the main source of circulating ketone bodies, contributing more than 90% to the total [12,13]. In hepatocytes, low glucose availability leads to ketogenesis and the release of ketone bodies that meet the energy demands of peripheral tissues, such as the brain, skeletal muscle, and heart (Figure 1B) [14]. When glucose availability is limited in these tissues, circulating ketone bodies serve as effective alternative energy substrates [15]. This metabolic shift is closely associated with improved metabolic flexibility, defined as the ability of an organism to adjust fuel oxidation between carbohydrates and lipids in response to substrate availability. Ketone bodies are subsequently oxidised in the mitochondria of those peripheral tissues [3,16]. Within the mitochondria of these tissues, ketone bodies undergo oxidation, generating acetyl-CoA, NADH, and FADH_2_, which then feed into the TCA cycle and oxidative phosphorylation to produce adenosine triphosphate (ATP) (Figure 1B).

In addition to serving as energy substrates, ketone bodies can exert effects via other mechanisms. Notably, βHB inhibits histone deacetylases (HDACs), thereby regulating gene expression associated with oxidative stress resistance and metabolic adaptation [17,18]. This includes upregulation of manganese superoxide dismutase (MnSOD) and catalase, as well as genes that support metabolic adaptation, such as PGC-1α [19]. As a result, βHB contributes to the metabolic advantages associated with a KD.

KDs are also linked to the regulation of nutrient sensing and cellular energy balance through activation of AMP-activated protein kinase (AMPK), inhibition of the mechanistic target of rapamycin (mTOR), and modulation of peroxisome proliferator-activated receptor (PPAR) signalling [20,21]. Collectively, these pathways promote mitochondrial biogenesis, improve cellular stress resilience, and enhance metabolic efficiency. In addition, ketone metabolism generates fewer reactive oxygen species (ROS) per unit of ATP than glucose metabolism, further supporting mitochondrial function and reducing oxidative stress [22,23].

Beyond metabolic regulation, β-hydroxybutyrate (βHB) has been shown to suppress NLRP3 inflammasome activation by preventing stimulus-induced K^+^ efflux, thereby maintaining intracellular ionic balance and inhibiting assembly of the active inflammasome complex [1,24]. Recent evidence also suggests that KD-induced alterations in cellular energy status may activate AMPK, leading to reduced stability of the immune checkpoint protein PD-L1 and increased expression of antigen presentation genes and type I interferon signalling [25]. Because PD-L1 normally limits excessive immune activation, its reduction may theoretically increase susceptibility to autoimmune responses [26]. However, the clinical relevance of these findings and their translation into human metabolic outcomes remain uncertain.

## 3. The Effects of a KD on Obesity and Diabetes

**Figure 2:**
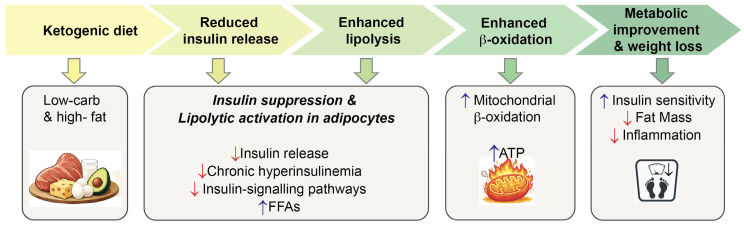
Proposed pathways of KD-induced weight loss and metabolic improvement. ↑, increase; ↓, decrease. A KD induces nutritional ketosis through carbohydrate restriction, lowering insulin secretion and increasing ketone body production. Enhanced adipose lipolysis raises circulating free fatty acids (FFAs), promoting mitochondrial fatty acid transport, β-oxidation, ketone utilisation, and ATP generation in skeletal muscle and liver. A KD reduces adipose inflammation by suppressing pro-inflammatory macrophage infiltration and inflammasome activation, thereby improving insulin sensitivity and adipocyte function. KD-associated weight loss improves peripheral insulin sensitivity (skeletal muscle and adipose tissue), whereas hepatic insulin sensitivity may improve less consistently.

Obesity is a major driver of metabolic dysfunction and insulin resistance. KDs have emerged as a promising intervention for overweight and obese individuals. Nutritional ketosis alters appetite regulation, substrate utilisation, and hormonal signalling, thereby facilitating reductions in body weight and fat mass (Figure 2) [3,15]. A KD influences endocrine regulation through hormonal responses essential for maintaining energy homeostasis and promoting ketone utilisation, particularly as the body shifts from glucose to fat as its primary energy substrate. Numerous studies in animal models and clinical trials report reductions in blood glucose, suggesting potential benefits for glycaemic control. The carbohydrate restriction inherent in a KD lowers circulating insulin levels, further reducing glucose uptake and glycogen synthesis in insulin-responsive tissues [3,27]. A KD with weight loss is associated with improved peripheral but not hepatic insulin sensitivity [28] (Figure 2).

Recent findings further suggest that a KD stimulates the secretion of gut hormones, such as glucagon-like peptide-1 (GLP-1) and peptide YY (PYY), both of which play significant roles in appetite regulation and insulin sensitivity [29,30,31]. Additionally, reduced insulin signalling, together with lower circulating insulin levels, leads to decreased leptin secretion from adipocytes, as insulin stimulates leptin secretion [32]. This decrease in leptin subsequently affects appetite control and energy balance. Like leptin, adiponectin, another adipokine secreted by adipocytes, regulates energy balance, metabolism, and inflammation, largely by modulating AMPK activity [33]. Indeed, adiponectin enhances insulin sensitivity and promotes fatty acid oxidation, while leptin acts as a satiety signal by suppressing appetite, thus supporting metabolic health [34,35]. Both hormones stimulate AMPK, which serves as a central regulator of cellular metabolism by coordinating catabolic and anabolic processes in relation to ATP generation or consumption, helping maintain cellular energy homeostasis. Moreover, βHB has been shown to reduce hypothalamic expression of SOCS3, a negative regulator of leptin signalling, and TNFα, an inflammatory marker linked to hypothalamic hormone resistance [36]. At the same time, βHB increased expression of POMC, an anorexigenic neuropeptide that suppresses appetite [36]. Use of ketone bodies, particularly βHB and AcAc, for cellular energy production via mitochondrial OXPHOS was significantly reduced in individuals with T2D or obesity and was associated with increased insulin resistance [37]. Impaired ketone utilisation in these individuals may reflect reduced mitochondrial oxidative capacity and metabolic inflexibility, with limited efficient substrate switching among glucose, fatty acids, and ketone bodies. Therefore, incomplete fatty acid oxidation and accumulation of lipidomic substances may disrupt insulin signalling pathways and further exacerbate insulin resistance [38].

The weight-loss effects of KDs have been consistently observed in both animal models and clinical studies. However, weight-loss efficiency may vary between individuals with and without T2D. Meta-analysis studies found no statistically significant difference in weight loss between people with and without T2D on a KD [39,40]. However, another study comparing people with T2D to those without has shown slightly less weight loss, which may be due to multiple factors such as hyperinsulinaemia, diabetic status, and the effects of diabetic medications [41]. These factors could potentially impact the success of weight loss, even with specialised dietary approaches. Therefore, individuals with T2D may receive therapeutic support, such as medications that improve insulin sensitivity, and reduce hypoglycaemia-causing drugs when commencing weight-loss diets. The details of studies on antidiabetic medications will be covered in the next section.

Additionally, interpreting the weight-loss effects of KDs is limited to determining whether the observed outcomes are directly attributable to ketone bodies or are secondary to other factors such as caloric restriction, carbohydrate reduction, macronutrient composition, or weight loss. For instance, improvements in glycaemic control and insulin sensitivity may partly reflect weight reduction [42]. In humans, the majority of glycaemic and insulin sensitivity improvements occur with energy restriction and weight loss; ketosis-specific effects have not been consistently demonstrated under weight-maintaining conditions. Similarly, changes in dietary fat and protein intake may independently influence inflammatory and metabolic pathways [43], thereby confounding the interpretation of KD-specific effects.

A recent study in carnosine dipeptidase 2 (CNDP2)-deficient mice provides critical mechanistic insight into the efficacy of KD in promoting weight loss [44]. In this study, KD increased circulating βHB levels, enabling CNDP2-dependent βHB-ylation of amino acids and the generation of bioactive βHB–amino acid metabolites [44]. Notably, despite substantial increases in circulating ketone bodies after KD, CNDP2-knockout mice exhibited a reduced anorexigenic response and higher body weight, whereas control mice did not [44]. These findings indicate that elevated ketone levels alone may be insufficient to mediate the metabolic benefits of KDs. Rather, CNDP2-dependent conversion of βHB into bioactive metabolites is a critical determinant of KD-induced appetite suppression and weight reduction. These findings suggest that ketosis-specific metabolic signalling pathways may contribute to the appetite-suppressive and metabolic effects of KDs beyond those of simple carbohydrate restriction, although the outcomes currently come from preclinical and animal studies. Currently, no human data confirm a CNDP2-dependent pathway in KD-treated individuals.

Given the growing evidence that ketone bodies may contribute to metabolic regulation, studies specifically investigating the direct effects of ketones, such as exogenous ketone administration, provide important complementary insights. Whereas KDs can induce metabolic adaptations over chronic time frames, exogenous ketones enable acute elevations in circulating ketones, allowing isolation of the immediate, ketone-specific effects on metabolic regulation. Consistent with this concept, a recent study directly tested whether acute elevations in circulating ketones would worsen glucose homeostasis via increased skeletal muscle ketone oxidation [45]. Oral administration of a ketone ester markedly increased circulating βHB levels and improved glucose tolerance in diet-induced obese mice, whereas no effect was observed in lean controls [45]. Complementary ex vivo experiments using isolated islets demonstrated that the oxidizable R-isomer of βHB elicited greater insulin secretion than the S-isomer in islets from obese mice [45], suggesting a potential direct effect of ketones on β-cell function. Nevertheless, it remains challenging to determine whether the effects are due to dietary change or to ketosis itself, given the gaps between preclinical and clinical studies and the lack of long-term studies.

A randomised clinical study involving 160 adults with obesity found the greatest weight loss in the very low-carbohydrate ketogenic diet group, even compared with a calorie-restricted Mediterranean diet [46]. Both the KD and modified alternate-day fasting groups showed the highest ketone levels, suggesting that nutritional ketosis may contribute to metabolic adaptations beyond simple caloric restriction [46]. However, despite greater weight reduction over 3 months, significant differences between groups in cardiometabolic parameters were limited, highlighting the need for longer-term studies to distinguish ketosis-specific effects from those mediated by weight loss [46]. Additional findings from human trials reported over the past five years are summarised in Table 1. These studies include healthy participants as well as those with overweight/obesity and T2D.

Recent studies on obesity and T2D, involving large cohorts and long-term KDs with subsequent longitudinal research, provide important clinical evidence demonstrating the sustainability of ketogenic interventions. In a randomised study involving over 160 patients with T2D who had been treated with diabetic medication for over 10 years, sustained improvements were observed over six months in diabetes medication use, HbA1c levels, time within the target blood glucose range, and body weight [47,48]. This suggests clinically meaningful metabolic benefits that are difficult to achieve with medication alone.

Furthermore, a one-year randomised study of overweight and obese adults demonstrated that a healthy KD—designed to address issues such as increased saturated fat intake, elevated LDL-C, fibre deficiency, and micronutrient deficiencies associated with traditional KDs—can effectively promote weight loss and improve cardiovascular and metabolic health without increasing LDL cholesterol levels [49]. To avoid potential risk factors, a KD includes low saturated fat, high unsaturated fat, adequate fibre, micronutrient supplementation, and controlled protein intake [49]. The findings suggest that a well-structured KD, along with continuous monitoring, supports its sustainability and potential therapeutic benefits in clinical practice.

**Table 1:** Overview of clinical studies.

Study and Type	Population	Interventions	Major Outcomes
Battezzati et al., Italy [50], RCrT	Healthy adults (n = 12)	Ketogenic meal vs. Mediterranean meal	Ketogenic meal reduced BGL, insulin and *C*-peptide levels.
Buga et al. [51], RCT	Overweight/obese adults (n = 37)	KD +/− ketone supplementation vs. low-fat diet	KD reduced BGL and insulin resistance (HOMA-IR). Ketone supplementation further reduced BGL and ketosis but not insulin resistance/insulin.
Merovci et al. [52], RCT	Adults with obesity and T2D (n = 29)	Standard diet and KD +/− ketone supplementation	KD lowered OGTT glucose without major changes in insulin sensitivity or body composition.
Gower et al., USA [53], RCT	Adults with T2D (n = 56)	KD vs. low-fat diet	KD increased β-hydroxybutyrate and reduced hepatic fat.
Gardner et al., USA [54], RCrT	Adults with prediabetes/T2D (n = 33)	Whole-food KD vs. Mediterranean diet	KD improved weight loss and triglycerides but increased LDL, while HbA1c remained unchanged.
Sanchez et al., Spain [55], RCT	Adults with obesity (n = 30)	VLCKD vs. Mediterranean diet	VLCKD reduced weight, BMI and inflammatory markers.
Willis et al., USA [48], parallel-group RCT	Adults with T2D (n = 163)	Medically supervised KD for 12 weeks	Sustained reduction in weight, HbA1c, glucose and diabetes medication use over 3 months.
Willis et al., USA [47], FU of RCT	Adults with T2D, (n = 163) 6-month FU of above subjects	Medically supervised KD for 24 weeks	Sustained reduction in weight, BMI, HbA1c (−1.3%), BGL, diabetes medication, energy and carbohydrate intake. Continued improvements.
Martinez-Montoro et al., Spain [46], RCT, comparative	Adults with obesity (n = 160)	KD vs. Mediterranean, ADF and TRE diets	KD produced greatest weight loss and improved glucose, HOMA-IR and triglycerides, but increased LDL.
Hall et al., USA [56], RCrT	Overweight adults (n = 20)	Low-carbohydrate vs. low-fat diet	Low-carbohydrate diet lowered triglycerides and *C*-peptide but worsened OGTT glucose.
Saslow et al., USA [57], RCT	Adults with obesity and prediabetes/T2D (n = 94)	VLCD vs. DASH diet	VLCD achieved greater weight loss and HbA1c reduction; DASH improved systolic BP more.
Guevara-Cruz et al., Mexico [58], RCT	Adults with obesity (n = 44)	Calorie restriction, IF, KD or ad libitum diet	KD reduced weight and fat mass and improved monocyte metabolic function and gut microbiota diversity.
Tay et al., Singapore [59], RCT	Adults with obesity (n = 50)	KD vs. KD with ready-to-eat meals	KD-ready-to-eat improved HbA1c cholesterol and BP more than standard KD.
Kackley et al., USA [60], RCT	Females with obesity (n = 19)	KD +/− ketone supplementation vs. low-fat diet	KD improved weight loss, body composition, and surrogate cardiometabolic parameters, including fasting insulin, HOMA-IR, and lipid profile.
Wachsmuth et al., Germany [61], RCrT	Healthy adults (n = 24)	Low-carb diet progressing to KD	KD reduced weight and fat mass and increased βHB without affecting cholesterol or triglycerides.
Tzenios et al., Canada [62], single-arm interventional study	Overweight adults (n = 14)	VLCKD cohort study	VLCKD reduced weight, BMI, body fat and HbA1c, but increased cholesterol and LDL.
Zhang et al., China [63], single-arm interventional study	Adults with obesity (n = 30)	Modified Chinese KD	KD improved weight, BMI and adiposity markers.
Gao et al., China [64], RCT	Adults with T2D (n = 104)	Dulaglutide +/− KD	Combination therapy improved glycaemic control, insulin sensitivity and lipid profiles.
Lim et al., Singapore [49], RCT	Adults with obesity (n = 80)	KD vs. energy-restricted diet	KD improved weight, BMI, BP and HbA1c over 12 months.
Li et al., China [65], RCT	Adults with overweight and newly diagnosed T2D (n = 60)	Control vs. KD	KD improved BMI, glucose control, insulin and lipid profile.
Kikuchi et al., Japan [66], RCT	Adults with overweight/obesity (n = 42)	Low-carb vs. very low-carb diet	Both diets reduced weight and improved lipids and liver function.
Luong et al., Denmark [28], RCrT	Adults with obesity (n = 11)	Standard diet vs. KD	KD reduced weight, TG and glucose and improved insulin sensitivity.
Mela et al., Spain [67], RCT	Adults with obesity (n = 96)	KD vs. Mediterranean vs. alternate-day fasting	KD reduced weight and BMI without affecting cognition or gut microbiota.
Du et al., USA [68], RCT	Adults with obesity (n = 60)	KD vs. low-fat diet	KD improved weight, BMI, HbA1c and BP, particularly at 3 months.

Where reported, nutritional ketosis was assessed by blood β-hydroxybutyrate concentrations and/or urinary ketone measurements. Not all studies included in this table objectively verified ketosis. Abbreviations: ADF, alternate-day fasting; BGL, blood glucose level; βHB, beta-hydroxybutyrate; BMI, body mass index; BP, blood pressure; DASH, dietary approaches to stop hypertension; FU, follow up; HbA1c, glycosylated haemoglobin; HOMA-IR, homeostatic model assessment of insulin resistance; IF, intermittent fasting; KD, ketogenic diet; LDL, low-density lipoprotein; OGTT, oral glucose tolerance test; RCT, Randomised Controlled Trial; RCrT, Randomised Cross-over Trial; TG, triglycerides; T2D, type 2 diabetes; TRE, time-restricted eating; VLCD, very low-carbohydrate diet; VLCKD, very low-carbohydrate ketogenic diet.

A KD is a double-edged intervention, conferring both metabolic benefits and harms that depend on its duration, metabolic context, and the host context targeted. For example, short-term KD interventions appear to elicit rapid, largely adaptive responses. In a clinical study, healthy individuals on a KD for 3 days showed improved insulin sensitivity, along with increased nutritional ketosis, circulating βHB, and fibroblast growth factor 21 (FGF21) [69]. Since increased FGF21 acts as a negative regulator of the NLRP3 inflammasome [70], it reduced the inflammatory response by inhibiting NLRP3 inflammasome activation and suppressing the release of pro-inflammatory cytokines [69,70]. Notably, a recent study in obese diabetic mouse models and patients with alcoholic fatty liver disease has shown that hepatic FGF21 responsiveness may contribute to some of the metabolic adaptations observed during KD intervention [71]. In particular, the circadian rhythm gene *BMAL1* is identified as a key regulator, along with FGF21, within the BMAL1–FGF21 axis, which leads to metabolic adaptation [71]. In both diabetic mice and these patients, even after KD treatment, impaired BMAL1 function, reduced FGF21 responsiveness, and lipid dysregulation were observed, indicating that FGF21 signalling is associated with several metabolic adaptations induced by KD [71]. These findings emphasise that the positive effects of a KD on obesity and diabetes may be associated with FGF21-linked pathways, including immune regulation and lipid metabolism. They also underscore the importance of considering the metabolic context and suggest that strategies targeting FGF21 may help optimise a KD’s therapeutic outcomes.

Consistent with this, an 8-day KD intervention reduced body weight, fat mass, liver weight, fasting glucose, and insulin levels, largely mimicking the effects of protein restriction in a mouse model [72]. These changes were also accompanied by distinct alterations in hepatic gene expression [72]. However, an extended KD appears to be associated with a progressive divergence between glycaemic control and lipid homeostasis. Indeed, in the T2D mouse model, 8 weeks of KD improved glucose and insulin tolerance, but promoted dyslipidaemia, increased adipose tissue mass, and significant hepatic lipid accumulation [73]. This raises concerns about long-term hepatic safety despite improved glycaemic control. A study by Gallop et al. [74] found that mice fed a KD for nearly 1 year developed severe hyperlipidaemia, hepatic dysfunction, marked glucose intolerance, insulin resistance and impaired insulin secretion. Notably, the extended KD induced ER and Golgi stress in pancreatic β-cells, disrupting insulin granule trafficking and secretory capacity.

However, the glycaemic reduction induced by a KD may directly reduce the workload on pancreatic β-cells, thereby improving their functionality. For instance, a clinical study in overweight or obese patients with metabolic hypogonadism demonstrated improved β-cell function following a KD [75]. After 12 weeks of intervention, proinsulin levels returned to the normal range, with no change in the proinsulin-to-insulin ratio. Indeed, proinsulin levels increase when β-cells are under stress or when secretory pressure is excessive [75]. These clinical outcomes indicate that the KD intervention primarily reduces the β-cell secretory load rather than correcting intrinsic proinsulin-processing defects. Additionally, a study by Furth-Lavi et al. [76] involving doxycycline-inducible β-cell-damaged mice shows that a 4-week KD intervention helps regenerate existing β-cells, thereby increasing β-cell mass and improving glucose regulation. The regeneration of existing β-cells is crucial for people with diabetes, and a KD may support this process. Overall, while a short-term KD promotes metabolic flexibility and anti-inflammatory signals, a prolonged KD induces cumulative lipid and secretory stress that may ultimately impair β-cell function and compromise glucose regulation, highlighting the importance of carefully considering dietary duration and composition in therapeutic settings.

### Clinical Case Reports and Preclinical Studies on KD Using Antidiabetic Medications

GLP-1 receptor agonists (GLP-1RAs) are approved medications for the treatment of obesity and T2D, with benefits in weight loss and improved glycaemic control [77]. Although current guidelines for combining GLP-1 RAs with a KD lack robust clinical evidence, some reports suggest synergistic effects [64,78]. For example, combining a KD with dulaglutide significantly improved glucose and lipid metabolism and enhances insulin sensitivity in 104 patients with T2D, as shown by lower blood glucose and lipid levels after six months of treatment [64].

SGLT-2 inhibitors, including canagliflozin, dapagliflozin, and empagliflozin, lower blood glucose by increasing urinary glucose excretion and reducing insulin demand, thereby alleviating metabolic stress on pancreatic β-cells [79,80,81]. Similarly to KD, these agents improve metabolic health through distinct mechanisms. KD enhances fatty acid oxidation and ketone-based energy metabolism, whereas SGLT-2 inhibitors reduce hyperglycaemia through renal glucose disposal. Dapagliflozin promotes WAT browning via the FGFR1–LKB1–AMPK pathway and modulates glucagon secretion by α-cells under high-glucose conditions, thereby improving energy metabolism and hormonal regulation [82,83]. Therefore, both KD and SGLT-2 inhibitors may offer a multifaceted approach to managing obesity and diabetes by supporting metabolic and hormonal balance through various pathways. Combining these applications can thus help maintain normal blood glucose levels by promoting renal glucose excretion and regulating endogenous glucose production.

Nevertheless, interactions between these pharmacological therapies and dietary interventions may lead to unexpected metabolic risks that vary among individuals. Severe diabetic ketoacidosis (DKA) has been reported in patients with T2D following initiation of a KD while concurrently being treated with SGLT-2 inhibitors [84,85]. Importantly, euglycaemic diabetic ketoacidosis (euDKA), characterised by severe acidosis with normal or mildly elevated blood glucose (typically less than 250 mg/dL), poses a significant risk of missed or delayed diagnosis of severe DKA in patients with T2D [84,86]. When combined with SGLT-2 inhibitors and a KD, this combination can precipitate ketogenesis beyond physiological adaptation, causing adverse effects, as reported in a recent case study of a patient with T2D [85]. In contrast, another case study of a woman with T2D reported that adverse outcomes are not necessarily intrinsic to ketogenic interventions [87]. In this case, a KD was successfully reintroduced after euDKA by discontinuing the SGLT-2 inhibitor, maintaining continuous metabolic monitoring, gradually reducing carbohydrate intake, and individualising insulin titration [87]. Nevertheless, caution is warranted when combining a KD with SGLT-2 inhibitors, as this combination has been linked to an increased risk of euDKA. Such interventions should be undertaken only under strict medical supervision with careful metabolic monitoring. Although the American Diabetes Association and the European Association for the Study of Diabetes guidelines do not provide explicit recommendations on intentionally combining a KD with SGLT-2 inhibitors [88,89], several expert consensus statements caution against this approach because of the increased risk of euDKA [90,91].

Although a gap remains between animal models and the clinical application of dietary interventions, preclinical findings could provide a mechanistic rationale for considering KD in combination with antidiabetic medications. Fujita Y. et al. [92] observed that Akita mice fed a KD in combination with the SGLT-2 inhibitor ipragliflozin for 8 weeks showed improved glycaemic control and partially preserved islet morphology, with an increased β-/α-cell area ratio, indicating synergistic benefits compared with untreated mice. This suggests interactions between antidiabetic drugs and diet may converge on islet architecture, beyond improved glucose regulation. Preclinical studies and large-scale clinical trials are required, however, to better understand the therapeutic potential of combining KDs with SGLT-2 inhibitors.

Given that chronic inflammation and cellular stress contribute to the progression of obesity and diabetes, emerging studies have begun to examine whether KDs influence cellular senescence pathways. In mice, KD-related senescence and circulating senescence-associated secretory phenotype (SASP) markers were reduced by senolytic drugs and altered by an intermittent KD [93]. Additionally, a study by M. Wakita et al. [94] found that KD-induced metabolic stress increased the vulnerability of senescent cells to senolytic therapies, likely by increasing mitochondrial workload and lowering resistance [94]. These observations are currently limited to preclinical mouse studies, so the human translational relevance remains uncertain.

**Figure 3:**
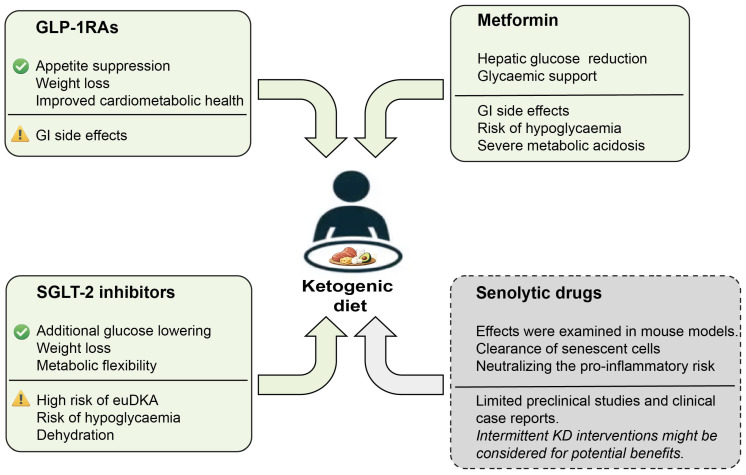
Potential benefits and risks of combining KD with antidiabetic medications. Schematic illustration of how KD affects health outcomes with different antidiabetic drugs. The effects of GLP-1RAs, metformin, and SGLT-2 inhibitors were assessed in both preclinical and clinical studies, whereas senolytic drugs were examined only in preclinical mouse models. Abbreviations: GI, gastrointestinal; euDKA, euglycaemic diabetic ketoacidosis.

Overall, these findings highlight that the risks and benefits of KD as a therapeutic strategy depend heavily on individual metabolic status, medication use, and medication interactions (Figure 3). These observations further support the emerging concept of precision nutrition in the management of obesity and T2D, in which dietary interventions are tailored to the metabolic context, pharmacological treatment, and ongoing clinical monitoring. However, despite the mechanistic promise of these integrated therapeutic approaches, current evidence remains limited and is derived predominantly from preclinical studies, small clinical cohorts, or case reports. Therefore, larger, well-controlled clinical trials are required before such strategies can be broadly implemented in routine clinical practice.

## 4. KD and Gut Health in Obesity and Diabetes

Gut health, as reflected in the gut microbiota, plays a vital role in linking host health outcomes directly to diet through nutrient sources, compositions, and proportions [95,96]. A comprehensive metabolomic study of obese individuals examined how a KD alters intestinal permeability [97]. Using urinary and faecal samples, the study linked changes in permeability to circulating interleukins and lipopolysaccharides, highlighting the role of low-grade inflammation in obesity-related conditions. In healthy adults, a 12-week KD impacted gut microbial diversity and skeletal muscle phenotype. While alpha diversity remained stable, the KD induced a significant shift in beta diversity, marked by a substantial and sustained decrease in *Bifidobacterium* and *Planococcus*. Simultaneously, skeletal muscle underwent metabolic reprogramming towards fat oxidation, as indicated by the induction of *PDK4* and the suppression of *INSR*, *AMPK*, *GLUT4*, and *PLIN* [98]. However, despite improvements at 4 weeks, fasting glucose was not improved at 12 weeks [98].

**Figure 4:**
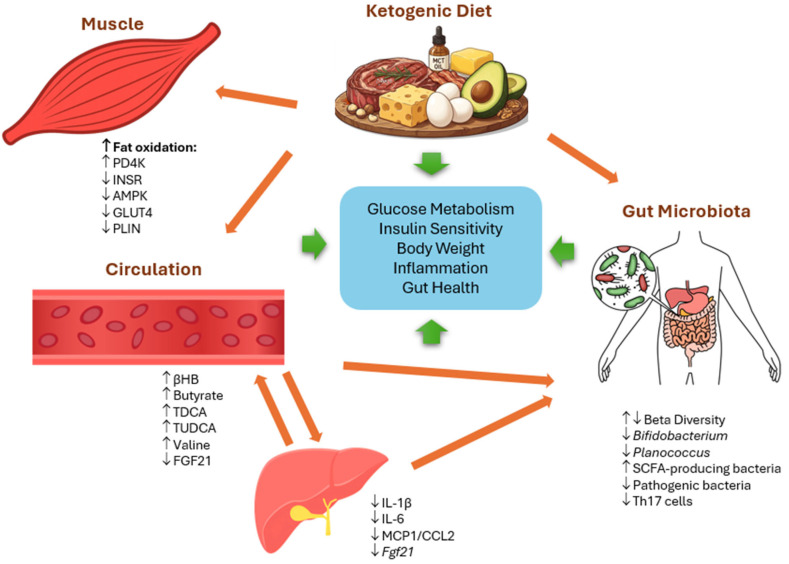
The mechanistic link between a KD, the gut microbiota, and metabolic health. A KD induces a significant shift in beta diversity within the gut microbiota, characterised by a substantial and sustained depletion of *Bifidobacterium* and *Planococcus*. Simultaneously, skeletal muscle undergoes metabolic reprogramming toward enhanced fat oxidation, as evidenced by the induction of *PDK4* and the suppression of *INSR*, *AMPK*, *GLUT4*, and *PLIN*. Within the intestinal environment, the KD-associated microbiota reduces the prevalence of pro-inflammatory Th17 cells. The KD metabolite butyrate increases the abundance of SCFA-producing bacteria while suppressing pathogenic populations, thereby reducing hepatic levels of IL-1β, IL-6, and MCP-1/CCL2. Furthermore, a KD elevates circulating levels of TDCA and TUDCA, both of which are correlated with reduced body weight and improved fasting glucose. Additionally, a KD modulates serum valine levels via microbial activity, thereby modulating hepatic Fgf21 expression and circulating FGF21 levels. Abbreviations: INSR, insulin receptor; PDK4, pyruvate dehydrogenase kinase 4; PLIN, perilipin family protein; SCFA, short-chain fatty acid; TDCA, taurodeoxycholic acid; TUDCA, tauroursodeoxycholic acid.

Ang et al. [99] observed significant changes in the gut microbiota and increased circulating ketone bodies in 17 overweight or obese, non-diabetic participants during a 4-week KD. Among the microbial changes, *Bifidobacterium*, particularly *B. adolescentis*, showed a marked reduction. Although *Bifidobacterium* is generally considered beneficial for human health, its decreased abundance during a KD may reflect altered substrate availability and ketone-driven microbial selection rather than unequivocally adverse dysbiosis. Mechanistically, the ketone body βHB appears to suppress the growth of specific bifidobacterial species through a dose-dependent, pH-mediated mechanism. Consistent with this, ketone ester supplementation under carbohydrate restriction also suppressed bifidobacterial growth in HFD-fed mice [99]. Notably, transplantation of the KD human microbiota into germ-free mice further reduced levels of pro-inflammatory Th17 cells in the gut [99]. In a mouse study by S. Zhai et al. [100], butyrate, a ketogenic metabolite produced through gut microbial fermentation, supported gut health by modulating the gut microbiota rather than through direct KD intervention. Butyrate treatment in mice eating an HFD increased the number of short-chain fatty acid-producing bacteria while lowering potentially pathogenic bacterial populations, with reduced IL-1β, IL-6, and MCP-1 (monocyte chemotactic protein-1)/CCL2 [100]. In contrast, Daïen and colleagues suggested that a diet low in microbiota-accessible carbohydrates may disrupt the gut microbiota, weaken the gut barrier, and impair immune regulation [101]. In particular, when dietary fibre availability is low, gut bacteria may shift toward degrading the intestinal mucus layer, promoting mucus-degrading bacteria, increasing gut permeability, and contributing to altered T cell responses, reduced IgA/IgG production, and impaired resolution of inflammation [101]. These findings suggest that a KD and its metabolites might influence immune regulation through microbiome-dependent mechanisms. The proposed interactions among KD, the gut microbiota, bile acid metabolism, and metabolic health are summarised in Figure 4. 

In addition to the ketone body-driven KD, a recent study highlighted the role of bile acid metabolism in the gut microbiota. X. Li et al. [102] identified a new gut microbiota-dependent mechanism that links KD consumption to metabolic benefits via changes in bile acid metabolism. In their mouse model, KD feeding increased circulating levels of taurine-conjugated bile acids, taurodeoxycholic acid (TDCA) and tauroursodeoxycholic acid (TUDCA). Mechanistically, KD induced remodelling of the gut microbiota, including a decrease in Lactobacillus murinus ASF361 [102]. Under normal conditions, this bacterium helps deconjugate taurine-conjugated bile acids (TDCA and TUDCA) [103,104]. However, KD-induced reduction in this bacterium leads to a decline in intestinal bile salt hydrolase activity, thereby permitting greater resorption of TDCA/TUDCA, higher circulating bile acids and improved metabolism [102]. These associations were also observed in people with overweight or obesity, in which increases in TDCA and TUDCA were associated with lower body weight and fasting glucose [102]. Overall, these findings support a potential interaction among KDs, the gut microbiota, and bile acid metabolism, although the metabolic consequences may vary depending on host and dietary context.

Given that the effects of a KD on the gut microbiota appear important for weight loss and glucose metabolism, a mouse model study aimed at uncovering these mechanisms confirmed that a KD interacts with the gut microbiota to alter serum valine levels [105]. This alteration was associated with changes in hepatic FGF21 expression, which may contribute to the regulation of body weight and glucose metabolism [105]. As described earlier, FGF21 is implicated in pathways regulating insulin sensitivity, energy expenditure, lipid metabolism, and inflammatory responses [106]. Another mouse study suggested that gut microbiota-associated activation of FGF21-related pathways may represent an adaptive stress response during dietary restriction, particularly under conditions of limited protein intake [107]. Altogether, these findings suggest a potential interaction between KDs, the gut microbiota, and FGF21 signalling in metabolic regulation. However, the long-term consequences of KD-induced microbial alterations, as inferred from preclinical studies, remain uncertain in clinical practice, as these changes may confer both adaptive and maladaptive metabolic adaptations in a host-dependent manner. Collectively, these studies suggest that FGF21 acts as a central node integrating a KD, the microbiota, and metabolic adaptation, but human data are limited. Moreover, human data on long-term KD-induced changes in the gut microbiota remain sparse and heterogeneous, limiting conclusions about their long-term metabolic and clinical significance.

Metformin is the usual first-line agent for treating T2D [108]. Interestingly, metformin increases circulating ketone levels, albeit only slightly, in healthy individuals [109]. Metformin impacts the gut microbiota [110]. For example, metagenomic analysis in T2D patients shows increased abundance of certain bacterial species with higher growth rates following metformin treatment [111]. This suggests that metformin promotes the growth of specific gut bacteria. Particularly, changes in the gut microbiota vary across dietary interventions, including KDs, indicating that diet influences bacterial composition and cellular metabolism [111]. Notably, bacterial species that increase after a KD produce proline, valine, and carnosine, amino acid metabolites potentially linked to obesity or depression-like states [96,112].

SGLT-2 inhibitors and KDs both increase endogenous ketone body production, raising the risk of euDKA, as described in the clinical case reports and preclinical studies section. Due to this increased risk, the adverse effects of ketoacidosis outweigh the potential benefits of combining SGLT-2 inhibitors with KD, although each approach separately lowers blood glucose levels. Nonetheless, studies on SGLT-2 inhibitors report that they alter the host microbiome, particularly reducing harmful bacteria [113]. These effects also help decrease diabetes complications, organ damage, and inflammatory responses [113]. However, the long-term safety and efficacy of this therapeutic approach remain to be fully established, and several expert consensus statements caution against intentionally combining KD with SGLT-2 inhibitors due to the increased risk of euDKA [90,91]. Therefore, caution may be warranted when considering concurrent use of a KD and SGLT-2 inhibitors. In some contexts, pharmacological treatment with SGLT-2 inhibitors combined with alternative metabolic strategies, such as short-chain fatty acid supplementation, may provide metabolic and gut-related benefits while potentially reducing the risk of excessive ketone accumulation and ketoacidosis.

## 5. Disadvantages and Limitations of KDs

Despite the potential metabolic benefits of a KD, several limitations and safety concerns should be considered. Due to the restrictive nature of KDs, long-term adherence remains a major challenge across multiple clinical settings. Studies in obesity and T2D populations have reported difficulties sustaining strict carbohydrate restriction over time, particularly because of dietary monotony, psychosocial burden, and meal preparation demands. In addition, carbohydrate restriction may compromise the intake of several essential micronutrients, including thiamine, folate, vitamin A, magnesium, iron, and iodine [114].

A KD adversely affects lipid profiles in many individuals; increasing total and LDL cholesterol [115]. While KD interventions often improve triglyceride levels and increase HDL cholesterol, some individuals show marked increases in LDL cholesterol and ApoB-containing lipoproteins. For example, a meta-analysis reported that a KD significantly increased total and LDL-cholesterol concentrations in normal-weight individuals compared with control diets [116]. Likewise, an umbrella review by Chen et al. [117] demonstrated that a KD may increase LDL-cholesterol, total cholesterol, and HDL-cholesterol. Although increases in HDL cholesterol may be metabolically favourable, elevated LDL and total cholesterol levels could potentially increase atherosclerotic and cardiovascular risks, particularly in individuals with pre-existing cardiovascular risk factors. In addition, KDs are associated with an increased risk of kidney stones, with an estimated incidence of approximately 6% [118]. Proposed mechanisms include mild metabolic acidosis, hypocitraturia, urine pH imbalance, and hypercalciuria.

Several limitations should also be considered when interpreting the current evidence surrounding KD interventions. Most clinical studies investigating KDs have been relatively short-term, leaving the long-term safety, sustainability, and clinical effectiveness of these dietary strategies uncertain. Furthermore, many mechanistic insights derive from animal models or highly controlled experimental conditions that may not fully reflect the heterogeneity of human dietary adherence, genetics, medication use, and lifestyle factors. Consequently, direct translation of preclinical findings into long-term human metabolic outcomes remains challenging. In addition, substantial heterogeneity exists among ketogenic dietary interventions in macronutrient composition, caloric restriction, achievement of ketosis, participant characteristics, and intervention duration, complicating direct comparisons across studies. Long-term randomised controlled trials in diverse human populations remain limited, and many exploratory or preclinical findings require further validation in larger and longer-term clinical studies. Future research is therefore essential to clarify the long-term safety, practicality, and therapeutic efficacy of ketogenic interventions in obesity and diabetes management.

## 6. Summary and Future Directions

KDs have gained significant attention as a dietary approach to improve metabolic health in individuals with obesity and diabetes. Clinical studies report that a KD typically results in 1–2 kg weight loss per month early on. Recent umbrella reviews and meta-analyses suggest that a KD typically produces approximately 5–10% weight loss within 3–6 months, with partial maintenance of approximately 7–12% at 12 months in some individuals [119,120]. However, the reduction usually plateaus over time. Furthermore, individual metabolic adaptations—such as β-cell function, medication use, and diabetes progression—as well as insulin resistance in responsive tissues are critical determinants of weight loss. In particular, individuals with T2D, especially those with aggressive disease, may show only modest weight loss. By promoting ketogenesis and elevating circulating ketone bodies, a KD induces profound metabolic adaptations that affect glucose regulation, lipid metabolism, and inflammatory signalling pathways. Emerging evidence also indicates that a KD can alter the composition of the gut microbiota and microbial metabolite production, thereby supporting metabolic regulation via the gut–metabolism axis. Overall, these findings highlight the complex and heterogeneous metabolic effects of KDs, although the long-term clinical implications remain incompletely understood.

Most clinical studies on KDs have been relatively or very short-term, leaving the long-term safety and viability of such dietary strategies uncertain. Similarly, many mechanistic insights discussed in this review derive from short-term interventions or preclinical studies based on rodent models, and their direct translatability to long-term human metabolic outcomes remains uncertain. Rodent studies often employ highly controlled dietary compositions and metabolic conditions that may not fully reflect the heterogeneity of human dietary adherence, genetics, medication use, and lifestyle factors.

When treating overweight, obesity, and diabetes caused by β-cell dysfunction, it is crucial to consider the effects on pancreatic islet function and neighbouring tissues when aiming for a synergistic effect with a KD, especially during ongoing medication. A deep understanding of how different pancreatic endocrine cells, including β-cells, respond to nutrient imbalances and stress under the low-carbohydrate, high-fat dietary conditions of KD, and how they interact with each other, is essential. Additionally, advances in continuous metabolic monitoring technologies, including systems capable of tracking both glucose and ketone dynamics, may further support safer and more individualised implementation of ketogenic interventions. Several scientific and clinical associations have recognised low-carbohydrate and ketogenic diets as potential nutritional strategies for managing obesity and diabetes. The American Diabetes Association recognises low-carbohydrate eating as a possible way to improve glycaemic control and reduce the need for glucose-lowering medication in selected individuals with diabetes, highlighting its potential benefits [88]. While not a classic ketogenic diet, Diabetes UK recommend a lower-carbohydrate diet with 50–130 g/day as a short-term option for adults with T2D, but not for those with T1D. In contrast, the European Association for the Study of Diabetes considers carbohydrate restriction a strategy for weight loss and glycaemic management, but emphasises the importance of individualised therapy, overall dietary quality, nutritional adequacy, and long-term sustainability, rather than endorsing specific macronutrient ratios [89]. Both current guidelines consider low-carbohydrate diets a potential option for some people with obesity and diabetes. Importantly, KD is not synonymous with all low-carbohydrate diets. Many low-carbohydrate dietary interventions, particularly those that are less restrictive or implemented intermittently, may not achieve sustained nutritional ketosis and therefore may produce different metabolic effects. To better inform clinical practice and policy, robust, long-term research is essential.

This review has several limitations. First, as a narrative review, it may not have captured all relevant publications, and formal systematic review methodologies were not applied. Second, substantial heterogeneity exists among ketogenic dietary interventions in macronutrient composition, caloric restriction, achievement of ketosis, participant characteristics, and intervention duration, complicating direct comparisons between studies. Third, many mechanistic findings are derived from animal models or short-term human interventions, while long-term randomised controlled trial data in humans remain limited. Consequently, interpretation of long-term clinical outcomes is constrained by species-specific metabolic differences and heterogeneous clinical conditions among individuals. Finally, the strength of evidence varies considerably across the literature, with many exploratory or preclinical findings requiring further validation in larger, longer-term human studies. Long-term implementation of KD may also require individualised dietary supervision to ensure appropriate dietary composition and micronutrient intake. Future studies examining ketone supplementation, rather than sustained carbohydrate restriction alone, may provide additional therapeutic insight.

## Data Availability

No new data was created or analysed in this study.

## Abbreviations

The following abbreviations are used in this manuscript:

AcAc	Acetoacetate
AMPK	AMP-activated protein kinase
ATP	Adenosine triphosphate
BGL	Blood glucose level
βHB	β-hydroxybutyrate
CNDP2	Carnosine dipeptidase 2
DKA	Diabetic ketoacidosis
euDKA	Euglycaemic diabetic ketoacidosis
FADH_2_	Flavin Adenine Dinucleotide
FAs	Fatty acids
FGF21	Fibroblast growth factor 21
FGFR1	Fibroblast growth factor receptor 1
FU	Follow up
GLP-1	Glucagon-like peptide-1
GLP-1RAs	GLP-1 receptor agonists
HDACs	Histone deacetylases
INSR	Insulin receptor
KD	Ketogenic diet
LCD	Long-chain triglyceride
MCT	Medium-chain triglyceride
MnSOD	Manganese superoxide dismutase
mTOR	Mechanistic target of rapamycin
NADH	Nicotinamide Adenine Dinucleotide
OXPHOS	Oxidative phosphorylation
PDK4	Pyruvate dehydrogenase kinase 4
PLIN	Perilipin family protein
PPAR	Peroxisome proliferator-activated receptor
PYY	Peptide YY
ROS	Reactive oxygen species
SASP	Senescence-associated secretory phenotype
SCOT	Succinyl-CoA:3-ketoacid CoA transferase
SGLT-2 inhibitors	Sodium–Glucose Transport 2 inhibitors
SKD	Standard ketogenic diet
T1D	Type 1 diabetes
T2D	Type 2 diabetes
TCA	Tricarboxylic acid
TDCA	Taurodeoxycholic acid
TUDCA	Tauroursodeoxycholic acid
VLCKD	Very low-calorie ketogenic diet
WAT	White adipose tissue

## References

[1] Youm Y.H., Nguyen K.Y., Grant R.W., Goldberg E.L., Bodogai M., Kim D., D’Agostino D., Planavsky N., Lupfer C., Kanneganti T.D. (2015). The ketone metabolite beta-hydroxybutyrate blocks NLRP3 inflammasome-mediated inflammatory disease. Nat. Med..

[2] Malinowska D., Zendzian-Piotrowska M. (2024). Ketogenic Diet: A Review of Composition Diversity, Mechanism of Action and Clinical Application. J. Nutr. Metab..

[3] Zhu H., Bi D., Zhang Y., Kong C., Du J., Wu X., Wei Q., Qin H. (2022). Ketogenic diet for human diseases: The underlying mechanisms and potential for clinical implementations. Signal Transduct. Target. Ther..

[4] Mudaliar S. (2023). The Evolution of Diabetes Treatment Through the Ages: From Starvation Diets to Insulin, Incretins, SGLT2-Inhibitors and Beyond. J. Indian Inst. Sci..

[5] Westman E.C., Yancy W.S., Humphreys M. (2006). Dietary treatment of diabetes mellitus in the pre-insulin era (1914–1922). Perspect. Biol. Med..

[6] Kim J.M. (2017). Ketogenic diet: Old treatment, new beginning. Clin. Neurophysiol. Pract..

[7] Kilian J., Szlezak D., Tyszka-Czochara M., Filipowicz-Popielarska E., Bronowicka-Adamska P. (2026). The Ketogenic Diet in Type 2 Diabetes and Obesity: A Narrative Review of Clinical Evidence. Nutrients.

[8] Daley S.F., Masood W., Annamaraju P., Khan Suheb M.Z. (2026). The Ketogenic Diet: Clinical Applications, Evidence-Based Indications, and Implementation.

[9] McDonald T.J.W., Cervenka M.C. (2019). Lessons learned from recent clinical trials of ketogenic diet therapies in adults. Curr. Opin. Clin. Nutr. Metab. Care.

[10] Martinez-Reyes I., Chandel N.S. (2020). Mitochondrial TCA cycle metabolites control physiology and disease. Nat. Commun..

[11] Fernandez-Verdejo R., Mey J.T., Ravussin E. (2023). Effects of ketone bodies on energy expenditure, substrate utilization, and energy intake in humans. J. Lipid Res..

[12] Newman J.C., Verdin E. (2014). Ketone bodies as signaling metabolites. Trends Endocrinol. Metab..

[13] Puchalska P., Crawford P.A. (2017). Multi-dimensional Roles of Ketone Bodies in Fuel Metabolism, Signaling, and Therapeutics. Cell Metab..

[14] Arthur G., Adenawoola M.I., Wahba S., Montgomery B.S., Stec D.E. (2025). Role of Liver-Derived Ketones, Hepatokines, and Metabolites in the Regulation of Renal Function. Kidney360.

[15] Puchalska P., Crawford P.A. (2021). Metabolic and Signaling Roles of Ketone Bodies in Health and Disease. Annu. Rev. Nutr..

[16] (2021). The Effects of Ketogenic Diet on the Cardiac Substrate Metabolism and Brain Perfusion. https://clinicaltrials.gov/study/NCT05012748.

[17] Kong G., Huang Z., Ji W., Wang X., Liu J., Wu X., Huang Z., Li R., Zhu Q. (2017). The Ketone Metabolite beta-Hydroxybutyrate Attenuates Oxidative Stress in Spinal Cord Injury by Suppression of Class I Histone Deacetylases. J. Neurotrauma.

[18] Shimazu T., Hirschey M.D., Newman J., He W., Shirakawa K., Le Moan N., Grueter C.A., Lim H., Saunders L.R., Stevens R.D. (2013). Suppression of oxidative stress by beta-hydroxybutyrate, an endogenous histone deacetylase inhibitor. Science.

[19] Chriett S., Dabek A., Wojtala M., Vidal H., Balcerczyk A., Pirola L. (2019). Prominent action of butyrate over beta-hydroxybutyrate as histone deacetylase inhibitor, transcriptional modulator and anti-inflammatory molecule. Sci. Rep..

[20] Grabacka M., Pierzchalska M., Dean M., Reiss K. (2016). Regulation of Ketone Body Metabolism and the Role of PPARalpha. Int. J. Mol. Sci..

[21] Luo Y., Li J., Yang Y., Yan L., Huang Z., Chen L., Zhao L., Wang J., Yang Y., Liu X. (2026). Ketogenic diet modulates AMPK-mTOR pathway in breast cancer. J. Nutr. Biochem..

[22] Prince A., Zhang Y., Croniger C., Puchowicz M. (2013). Oxidative metabolism: Glucose versus ketones. Adv. Exp. Med. Biol..

[23] Board M., Lopez C., van den Bos C., Callaghan R., Clarke K., Carr C. (2017). Acetoacetate is a more efficient energy-yielding substrate for human mesenchymal stem cells than glucose and generates fewer reactive oxygen species. Int. J. Biochem. Cell Biol..

[24] Karmakar M., Katsnelson M.A., Dubyak G.R., Pearlman E. (2016). Neutrophil P2X7 receptors mediate NLRP3 inflammasome-dependent IL-1beta secretion in response to ATP. Nat. Commun..

[25] Dai X., Bu X., Gao Y., Guo J., Hu J., Jiang C., Zhang Z., Xu K., Duan J., He S. (2021). Energy status dictates PD-L1 protein abundance and anti-tumor immunity to enable checkpoint blockade. Mol. Cell.

[26] Zhang P., Wang Y., Miao Q., Chen Y. (2023). The therapeutic potential of PD-1/PD-L1 pathway on immune-related diseases: Based on the innate and adaptive immune components. Biomed. Pharmacother..

[27] Foley P.J. (2021). Effect of low carbohydrate diets on insulin resistance and the metabolic syndrome. Curr. Opin. Endocrinol. Diabetes Obes..

[28] Luong T.V., Pedersen M.G.B., Abild C.B., Lauritsen K.M., Kjærulff M.L.G., Møller N., Gormsen L.C., Søndergaard E. (2024). A 3-Week Ketogenic Diet Increases Skeletal Muscle Insulin Sensitivity in Individuals With Obesity: A Randomized Controlled Crossover Trial. Diabetes.

[29] Liu C., Ren N., Zhang H., Ma J. (2026). The role of PYY in improving insulin resistance. Front. Endocrinol..

[30] Liu C., Liu Y., Xin Y., Wang Y. (2022). Circadian secretion rhythm of GLP-1 and its influencing factors. Front. Endocrinol..

[31] Hengist A., Sciarrillo C.M., Guo J., Walter M., Hall K.D. (2024). Gut-derived appetite hormones do not explain energy intake differences in humans following low-carbohydrate versus low-fat diets. Obesity.

[32] Marques-Oliveira G.H., Silva T.M., Lima W.G., Valadares H.M.S., Chaves V.E. (2018). Insulin as a hormone regulator of the synthesis and release of leptin by white adipose tissue. Peptides.

[33] Khoramipour K., Chamari K., Hekmatikar A.A., Ziyaiyan A., Taherkhani S., Elguindy N.M., Bragazzi N.L. (2021). Adiponectin: Structure, Physiological Functions, Role in Diseases, and Effects of Nutrition. Nutrients.

[34] Xie L., O’Reilly C.P., Chapes S.K., Mora S. (2008). Adiponectin and leptin are secreted through distinct trafficking pathways in adipocytes. Biochim. Biophys. Acta.

[35] Baldelli S., Aiello G., Mansilla Di Martino E., Campaci D., Muthanna F.M.S., Lombardo M. (2024). The Role of Adipose Tissue and Nutrition in the Regulation of Adiponectin. Nutrients.

[36] Xu R., Takahashi N., Kaneko K. (2026). Ketone Body beta-Hydroxybutyrate Enhances Hypothalamic Leptin and Insulin Responsiveness. Nutrients.

[37] Zweck E., Piel S., Schmidt J.W., Scheiber D., Schon M., Kahl S., Burkart V., Dewidar B., Remus R., Chadt A. (2025). Impaired mitochondrial ketone body oxidation in insulin resistant states. EBioMedicine.

[38] Mir S.A., Narasimhan K., Annadurai J.K., Vaitheeswari, Ji S., Cameron-Smith D., Eriksson J.G., Leow M.K., Wenk M.R., Torta F. (2025). Lipidomic Signatures of Insulin Resistance Identified From Hyperinsulinemic-Euglycemic Clamp Studies in Asian Men. Diabetes.

[39] Leslie W.S., Taylor R., Harris L., Lean M.E. (2017). Weight losses with low-energy formula diets in obese patients with and without type 2 diabetes: Systematic review and meta-analysis. Int. J. Obes..

[40] Choy K.Y.C., Louie J.C.Y. (2023). The effects of the ketogenic diet for the management of type 2 diabetes mellitus: A systematic review and meta-analysis of recent studies. Diabetes Metab. Syndr..

[41] Bays H.E. (2023). Why does type 2 diabetes mellitus impair weight reduction in patients with obesity? A review. Obes. Pillars.

[42] Schenk S., Harber M.P., Shrivastava C.R., Burant C.F., Horowitz J.F. (2009). Improved insulin sensitivity after weight loss and exercise training is mediated by a reduction in plasma fatty acid mobilization, not enhanced oxidative capacity. J. Physiol..

[43] Shahamati D., Akhavan N.S., Rosenkranz S.K. (2025). Postprandial Inflammation in Obesity: Dietary Determinants, Adipose Tissue Dysfunction and the Gut Microbiome. Biomolecules.

[44] Moya-Garzon M.D., Wang M., Li V.L., Lyu X., Wei W., Tung A.S., Raun S.H., Zhao M., Coassolo L., Islam H. (2025). A beta-hydroxybutyrate shunt pathway generates anti-obesity ketone metabolites. Cell.

[45] Tabatabaei Dakhili S.A., Yang K., Locatelli C.A.A., Saed C.T., Greenwell A.A., Chan J.S.F., Chahade J.J., Scharff J., Al-Imarah S., Eaton F. (2023). Ketone ester administration improves glycemia in obese mice. Am. J. Physiol. Cell Physiol..

[46] Martinez-Montoro J.I., Bandera B., Gutierrez-Bedmar M., Gomez-Perez A.M., Macias-Gonzalez M., Moreno-Indias I., Tinahones F.J. (2025). Effect of a ketogenic diet, time-restricted eating, or alternate-day fasting on weight loss in adults with obesity: A randomized clinical trial. BMC Med..

[47] Willis H.J., Asche S.E., Adams R.N., Roberts C.G.P., McKenzie A.L., Krizka S., Athinarayanan S.J., Zoller A.R., Volk B.M., Bergenstal R.M. (2025). Effects of Continuous Glucose Monitoring Versus Blood Glucose Monitoring During a Carbohydrate-Restricted Nutrition Intervention in People With Type 2 Diabetes: 6-Month Follow-up Outcomes From a Randomized Clinical Trial. Endocr. Pract..

[48] Willis H.J., Asche S.E., McKenzie A.L., Adams R.N., Roberts C.G.P., Volk B.M., Krizka S., Athinarayanan S.J., Zoller A.R., Bergenstal R.M. (2025). Impact of Continuous Glucose Monitoring Versus Blood Glucose Monitoring to Support a Carbohydrate-Restricted Nutrition Intervention in People with Type 2 Diabetes. Diabetes Technol. Ther..

[49] Lim S.L., Tay M., Ang S.M., Wai S.N., Ong K.W., Neo W.J., Yap Q.V., Chan Y.H., Khoo C.M. (2024). Development and Pragmatic Randomized Controlled Trial of Healthy Ketogenic Diet Versus Energy-Restricted Diet on Weight Loss in Adults with Obesity. Nutrients.

[50] Battezzati A., Foppiani A., Leone A., De Amicis R., Spadafranca A., Mari A., Bertoli S. (2023). Acute Insulin Secretory Effects of a Classic Ketogenic Meal in Healthy Subjects: A Randomized Cross-Over Study. Nutrients.

[51] Buga A., Kackley M.L., Crabtree C.D., Bedell T.N., Robinson B.T., Stoner J.T., Decker D.D., Hyde P.N., LaFountain R.A., Brownlow M.L. (2023). Fasting and diurnal blood ketonemia and glycemia responses to a six-week, energy-controlled ketogenic diet, supplemented with racemic R/S-BHB salts. Clin. Nutr. ESPEN.

[52] Merovci A., Finley B., Hansis-Diarte A., Neppala S., Abdul-Ghani M.A., Cersosimo E., Triplitt C., DeFronzo R.A. (2024). Effect of weight-maintaining ketogenic diet on glycemic control and insulin sensitivity in obese T2D subjects. BMJ Open Diabetes Res. Care.

[53] Gower B.A., Yurchishin M.L., Goss A.M., Knight J., Garvey W.T. (2025). Beneficial Effects of Carbohydrate Restriction in Type 2 Diabetes Can Be Traced to Changes in Hepatic Metabolism. J. Clin. Endocrinol. Metab..

[54] Gardner C.D., Landry M.J., Perelman D., Petlura C., Durand L.R., Aronica L., Crimarco A., Cunanan K.M., Chang A., Dant C.C. (2022). Effect of a ketogenic diet versus Mediterranean diet on glycated hemoglobin in individuals with prediabetes and type 2 diabetes mellitus: The interventional Keto-Med randomized crossover trial. Am. J. Clin. Nutr..

[55] Sanchez E., Santos M.D., Nunez-Garcia M., Bueno M., Sajoux I., Yeramian A., Lecube A. (2021). Randomized Clinical Trial to Evaluate the Morphological Changes in the Adventitial Vasa Vasorum Density and Biological Markers of Endothelial Dysfunction in Subjects with Moderate Obesity Undergoing a Very Low-Calorie Ketogenic Diet. Nutrients.

[56] Hall K.D., Guo J., Courville A.B., Boring J., Brychta R., Chen K.Y., Darcey V., Forde C.G., Gharib A.M., Gallagher I. (2021). Effect of a plant-based, low-fat diet versus an animal-based, ketogenic diet on ad libitum energy intake. Nat. Med..

[57] Saslow L.R., Jones L.M., Sen A., Wolfson J.A., Diez H.L., O’Brien A., Leung C.W., Bayandorian H., Daubenmier J., Missel A.L. (2023). Comparing Very Low-Carbohydrate vs DASH Diets for Overweight or Obese Adults With Hypertension and Prediabetes or Type 2 Diabetes: A Randomized Trial. Ann. Fam. Med..

[58] Guevara-Cruz M., Hernandez-Gomez K.G., Condado-Huerta C., Gonzalez-Salazar L.E., Pena-Flores A.K., Pichardo-Ontiveros E., Serralde-Zuniga A.E., Sanchez-Tapia M., Maya O., Medina-Vera I. (2024). Intermittent fasting, calorie restriction, and a ketogenic diet improve mitochondrial function by reducing lipopolysaccharide signaling in monocytes during obesity: A randomized clinical trial. Clin. Nutr..

[59] Tay M.H.J., Yap Q.V., Lim S.L., Ong Y.W.Y., Wee V., Khoo C.M. (2025). The Effect of Short-Term Healthy Ketogenic Diet Ready-To-Eat Meals Versus Healthy Ketogenic Diet Counselling on Weight Loss in Overweight Adults: A Pilot Randomized Controlled Trial. Nutrients.

[60] Kackley M.L., Buga A., Brownlow M.L., O’Connor A., Sapper T.N., Crabtree C.D., Robinson B.T., Stoner J.T., Decker D.D., Soma L. (2024). Self-reported menses physiology is positively modulated by a well-formulated, energy-controlled ketogenic diet vs. low fat diet in women of reproductive age with overweight/obesity. PLoS ONE.

[61] Wachsmuth N.B., Aberer F., Haupt S., Schierbauer J.R., Zimmer R.T., Eckstein M.L., Zunner B., Schmidt W., Niedrist T., Sourij H. (2022). The Impact of a High-Carbohydrate/Low Fat vs. Low-Carbohydrate Diet on Performance and Body Composition in Physically Active Adults: A Cross-Over Controlled Trial. Nutrients.

[62] Tzenios N., Lewis E.D., Crowley D.C., Chahine M., Evans M. (2022). Examining the Efficacy of a Very-Low-Carbohydrate Ketogenic Diet on Cardiovascular Health in Adults with Mildly Elevated Low-Density Lipoprotein Cholesterol in an Open-Label Pilot Study. Metab. Syndr. Relat. Disord..

[63] Zhang N., Liu N., Zhao G., Yan J., Zhang P., Li X., Zhou J. (2025). Effects of a two-week modified ketogenic diet on circulating lipoprotein subclasses, GDF15, and FGF21 in obese adults. J. Transl. Med..

[64] Gao R., Yan L., Du X., He S., Xue L., Li T., Wei M., Gu Y. (2025). Effect of ketogenic diet plus dulaglutide on glucose and lipid metabolism in diabetes mellitus. Pak. J. Pharm. Sci..

[65] Li S., Lin G., Chen J., Chen Z., Xu F., Zhu F., Zhang J., Yuan S. (2022). The effect of periodic ketogenic diet on newly diagnosed overweight or obese patients with type 2 diabetes. BMC Endocr. Disord..

[66] Kikuchi T., Kushiyama A., Yanai M., Kashiwado C., Seto T., Kasuga M. (2023). Comparison of Weight Reduction, Change in Parameters and Safety of a Very Low Carbohydrate Diet in Comparison to a Low Carbohydrate Diet in Obese Japanese Subjects with Metabolic Disorders. Nutrients.

[67] Mela V., Heras V., Iesmantaite M., Garcia-Martin M.L., Bernal M., Posligua-Garcia J.D., Subiri-Verdugo A., Martinez-Montoro J.I., Gomez-Perez A.M., Bandera B. (2025). Microbiota fasting-related changes ameliorate cognitive decline in obesity and boost ex vivo microglial function through the gut-brain axis. Gut.

[68] Du Y., Wang J., Li S., Meireles C., Saliba A., Castillo A., Goros M., Gelfond J., Choi B.Y., Qi L. (2025). Digitally enhanced ketogenic diet versus low-fat diet for cardio-renal-metabolic health in a predominantly Hispanic adult population with overweight or obesity: Pilot randomised clinical trial. Diabetes Obes. Metab..

[69] Kim E.R., Kim S.R., Cho W., Lee S.G., Kim S.H., Kim J.H., Choi E., Kim J.H., Yu J.W., Lee B.W. (2022). Short Term Isocaloric Ketogenic Diet Modulates NLRP3 Inflammasome Via B-hydroxybutyrate and Fibroblast Growth Factor 21. Front. Immunol..

[70] Zeng Z., Zheng Q., Chen J., Tan X., Li Q., Ding L., Zhang R., Lin X. (2020). FGF21 mitigates atherosclerosis via inhibition of NLRP3 inflammasome-mediated vascular endothelial cells pyroptosis. Exp. Cell Res..

[71] Yang M., Guo Y., Du J., Li C., Bai H., Guo Y., Tan Y., Li X., Ren D., Li J. (2026). Impaired hepatic BMAL1-FGF21 signaling drives adverse metabolic outcomes of ketogenic diet. Life Sci..

[72] Kalafut K.C., Mitchell S.J., MacArthur M.R., Mitchell J.R. (2022). Short-Term Ketogenic Diet Induces a Molecular Response That Is Distinct From Dietary Protein Restriction. Front. Nutr..

[73] Zhang X., Qin J., Zhao Y., Shi J., Lan R., Gan Y., Ren H., Zhu B., Qian M., Du B. (2016). Long-term ketogenic diet contributes to glycemic control but promotes lipid accumulation and hepatic steatosis in type 2 diabetic mice. Nutr. Res..

[74] Gallop M.R., Vieira R.F.L., Mower P.D., Matsuzaki E.T., Liou W., Smart F.E., Roberts S., Evason K.J., Holland W.L., Chaix A. (2025). A long-term ketogenic diet causes hyperlipidemia, liver dysfunction, and glucose intolerance from impaired insulin secretion in mice. Sci. Adv..

[75] La Vignera S., Cannarella R., Galvano F., Grillo A., Aversa A., Cimino L., Magagnini C.M., Mongioi L.M., Condorelli R.A., Calogero A.E. (2021). The ketogenic diet corrects metabolic hypogonadism and preserves pancreatic ss-cell function in overweight/obese men: A single-arm uncontrolled study. Endocrine.

[76] Furth-Lavi J., Hija A., Tornovsky-Babeay S., Mazouz A., Dahan T., Stolovich-Rain M., Klochendler A., Dor Y., Avrahami D., Glaser B. (2022). Glycemic control releases regenerative potential of pancreatic beta cells blocked by severe hyperglycemia. Cell Rep..

[77] Lee C.Y. (2021). A Combination of Glucagon-Like Peptide-1 Receptor Agonist and Dietary Intervention Could Be a Promising Approach for Obesity Treatment. Front. Endocrinol..

[78] Camajani E., Masi D., Spizzichini M.L., Cori C., Rossetti R., Spoltore M.E., Tuccinardi D., Lubrano C., Gnessi L., Isidori A.M. (2025). Very low-calorie ketogenic diet and liraglutide as a synergistic strategy for the treatment of obesity: A short-term, non-randomised, observational, real-world clinical evaluation. Diabetes Obes. Metab..

[79] Poole R.M., Dungo R.T. (2014). Ipragliflozin: First global approval. Drugs.

[80] Chow E., Clement S., Garg R. (2023). Euglycemic diabetic ketoacidosis in the era of SGLT-2 inhibitors. BMJ Open Diabetes Res. Care.

[81] Asahara S.I., Ogawa W. (2019). SGLT2 inhibitors and protection against pancreatic beta cell failure. Diabetol. Int..

[82] Lv Y., Zhao C., Jiang Q., Rong Y., Ma M., Liang L., Li W., Zhang J., Xu N., Wu H. (2024). Dapagliflozin promotes browning of white adipose tissue through the FGFR1-LKB1-AMPK signaling pathway. Mol. Biol. Rep..

[83] Pedersen M.G., Ahlstedt I., El Hachmane M.F., Gopel S.O. (2016). Dapagliflozin stimulates glucagon secretion at high glucose: Experiments and mathematical simulations of human A-cells. Sci. Rep..

[84] Steinmetz-Wood S., Gilbert M., Menson K. (2020). A Case of Diabetic Ketoacidosis in a Patient on an SGLT2 Inhibitor and a Ketogenic Diet: A Critical Trio Not to Be Missed. Case Rep. Endocrinol..

[85] Guirguis H., Beroukhim Afrahimi S., Pham C. (2022). The Use of SGLT-2 Inhibitors Coupled With a Strict Low-Carbohydrate Diet: A Set-Up for Inducing Severe Diabetic Ketoacidosis. Clin. Med. Insights Case Rep..

[86] Nasa P., Chaudhary S., Shrivastava P.K., Singh A. (2021). Euglycemic diabetic ketoacidosis: A missed diagnosis. World J. Diabetes.

[87] Fieger E.I., Fadel K.M., Modarres A.H., Wickham E.P., Wolver S.E. (2020). Successful Reimplementation of a Very Low Carbohydrate Ketogenic Diet after Sglt2 Inhibitor Associated Euglycemic Diabetic Ketoacidosis. AACE Clin. Case Rep..

[88] Evert A.B., Dennison M., Gardner C.D., Garvey W.T., Lau K.H.K., MacLeod J., Mitri J., Pereira R.F., Rawlings K., Robinson S. (2019). Nutrition Therapy for Adults With Diabetes or Prediabetes: A Consensus Report. Diabetes Care.

[89] (2023). The Diabetes and Nutrition Study Group (DNSG) of the European Association for the Study of Diabetes (EASD). Evidence-based European recommendations for the dietary management of diabetes. Diabetologia.

[90] Umpierrez G.E., Davis G.M., ElSayed N.A., Fadini G.P., Galindo R.J., Hirsch I.B., Klonoff D.C., McCoy R.G., Misra S., Gabbay R.A. (2024). Hyperglycemic Crises in Adults With Diabetes: A Consensus Report. Diabetes Care.

[91] Danne T., Garg S., Peters A.L., Buse J.B., Mathieu C., Pettus J.H., Alexander C.M., Battelino T., Ampudia-Blasco F.J., Bode B.W. (2019). International Consensus on Risk Management of Diabetic Ketoacidosis in Patients With Type 1 Diabetes Treated With Sodium-Glucose Cotransporter (SGLT) Inhibitors. Diabetes Care.

[92] Fujita Y., Atageldiyeva K.K., Takeda Y., Yanagimachi T., Makino Y., Haneda M. (2020). A Low-Carbohydrate Diet Improves Glucose Metabolism in Lean Insulinopenic Akita Mice Along With Sodium-Glucose Cotransporter 2 Inhibitor. Front. Endocrinol..

[93] Wei S.J., Schell J.R., Chocron E.S., Varmazyad M., Xu G., Chen W.H., Martinez G.M., Dong F.F., Sreenivas P., Trevino R. (2024). Ketogenic diet induces p53-dependent cellular senescence in multiple organs. Sci. Adv..

[94] Wakita M., Ito K., Fujii K., Sakamoto D., Mikawa T., Sugawara S., Zhou X., Park J.H., Miyagawa H., Motooka D. (2026). Comparative analysis of senolytic drugs reveals mitochondrial determinants of efficacy and resistance. Nat. Aging.

[95] Li Y., Yang X., Zhang J., Jiang T., Zhang Z., Wang Z., Gong M., Zhao L., Zhang C. (2021). Ketogenic Diets Induced Glucose Intolerance and Lipid Accumulation in Mice with Alterations in Gut Microbiota and Metabolites. mBio.

[96] Galvez-Ontiveros Y., Paez S., Monteagudo C., Rivas A. (2020). Endocrine Disruptors in Food: Impact on Gut Microbiota and Metabolic Diseases. Nutrients.

[97] Calabrese F.M., Celano G., Riezzo G., D’Attoma B., Ignazzi A., Di Chito M., Sila A., De Nucci S., Rinaldi R., Linsalata M. (2023). Metabolomic Profiling of Obese Patients with Altered Intestinal Permeability Undergoing a Very Low-Calorie Ketogenic Diet. Nutrients.

[98] Hengist A., Davies R.G., Walhin J.P., Buniam J., Merrell L.H., Rogers L., Bradshaw L., Moreno-Cabanas A., Rogers P.J., Brunstrom J.M. (2024). Ketogenic diet but not free-sugar restriction alters glucose tolerance, lipid metabolism, peripheral tissue phenotype, and gut microbiome: RCT. Cell Rep. Med..

[99] Ang Q.Y., Alexander M., Newman J.C., Tian Y., Cai J., Upadhyay V., Turnbaugh J.A., Verdin E., Hall K.D., Leibel R.L. (2020). Ketogenic Diets Alter the Gut Microbiome Resulting in Decreased Intestinal Th17 Cells. Cell.

[100] Zhai S., Qin S., Li L., Zhu L., Zou Z., Wang L. (2019). Dietary butyrate suppresses inflammation through modulating gut microbiota in high-fat diet-fed mice. FEMS Microbiol. Lett..

[101] Daien C.I., Pinget G.V., Tan J.K., Macia L. (2017). Detrimental Impact of Microbiota-Accessible Carbohydrate-Deprived Diet on Gut and Immune Homeostasis: An Overview. Front. Immunol..

[102] Li X., Yang J., Zhou X., Dai C., Kong M., Xie L., Liu C., Liu Y., Li D., Ma X. (2024). Ketogenic diet-induced bile acids protect against obesity through reduced calorie absorption. Nat. Metab..

[103] Rowe J.C., Winston J.A. (2024). Collaborative Metabolism: Gut Microbes Play a Key Role in Canine and Feline Bile Acid Metabolism. Vet. Sci..

[104] Van den Bossche L., Hindryckx P., Devisscher L., Devriese S., Van Welden S., Holvoet T., Vilchez-Vargas R., Vital M., Pieper D.H., Vanden Bussche J. (2017). Ursodeoxycholic Acid and Its Taurine- or Glycine-Conjugated Species Reduce Colitogenic Dysbiosis and Equally Suppress Experimental Colitis in Mice. Appl. Environ. Microbiol..

[105] Wei X., Lu Y., Hong S. (2024). Gut Microbiota Modulates Fgf21 Expression and Metabolic Phenotypes Induced by Ketogenic Diet. Nutrients.

[106] Gliniak C.M., Gordillo R., Youm Y.H., Lin Q., Crewe C., Zhang Z., Field B.C., Fujikawa T., Virostek M., Zhao S. (2025). FGF21 promotes longevity in diet-induced obesity through metabolic benefits independent of growth suppression. Cell Metab..

[107] Martin A., Ecklu-Mensah G., Ha C.W.Y., Hendrick G., Layman D.K., Gilbert J., Devkota S. (2021). Gut microbiota mediate the FGF21 adaptive stress response to chronic dietary protein-restriction in mice. Nat. Commun..

[108] Gunton J.E., Cheung N.W., Davis T.M., Zoungas S., Colagiuri S., Australian Diabetes S. (2014). A new blood glucose management algorithm for type 2 diabetes: A position statement of the Australian Diabetes Society. Med. J. Aust..

[109] Dahabiyeh L.A., Mujammami M., Arafat T., Benabdelkamel H., Alfadda A.A., Abdel Rahman A.M. (2021). A Metabolic Pattern in Healthy Subjects Given a Single Dose of Metformin: A Metabolomics Approach. Front. Pharmacol..

[110] Wang Y., Jia X., Cong B. (2024). Advances in the mechanism of metformin with wide-ranging effects on regulation of the intestinal microbiota. Front. Microbiol..

[111] Ezzamouri B., Rosario D., Bidkhori G., Lee S., Uhlen M., Shoaie S. (2023). Metabolic modelling of the human gut microbiome in type 2 diabetes patients in response to metformin treatment. npj Syst. Biol. Appl..

[112] Mayneris-Perxachs J., Castells-Nobau A., Arnoriaga-Rodriguez M., Martin M., de la Vega-Correa L., Zapata C., Burokas A., Blasco G., Coll C., Escrichs A. (2022). Microbiota alterations in proline metabolism impact depression. Cell Metab..

[113] Hasanvand A., Goudarzi G., Hadian B. (2025). Pharmacological modulation of the diabetic gut microbiome with gliflozin drugs:new insights for therapeutic targeting. J. Diabetes Metab. Disord..

[114] Churuangsuk C., Griffiths D., Lean M.E.J., Combet E. (2019). Impacts of carbohydrate-restricted diets on micronutrient intakes and status: A systematic review. Obes. Rev..

[115] Li D., Dawson J., Gunton J.E. (2024). Therapeutic Potential of Ketogenic Interventions for Autosomal-Dominant Polycystic Kidney Disease: A Systematic Review. Nutrients.

[116] Joo M., Moon S., Lee Y.S., Kim M.G. (2023). Effects of very low-carbohydrate ketogenic diets on lipid profiles in normal-weight (body mass index <25 kg/m^2^) adults: A meta-analysis. Nutr. Rev..

[117] Chen S., Su X., Feng Y., Li R., Liao M., Fan L., Liu J., Chen S., Zhang S., Cai J. (2023). Ketogenic Diet and Multiple Health Outcomes: An Umbrella Review of Meta-Analysis. Nutrients.

[118] Acharya P., Acharya C., Thongprayoon C., Hansrivijit P., Kanduri S.R., Kovvuru K., Medaura J., Vaitla P., Garcia Anton D.F., Mekraksakit P. (2021). Incidence and Characteristics of Kidney Stones in Patients on Ketogenic Diet: A Systematic Review and Meta-Analysis. Diseases.

[119] Bachar A., Birk R. (2025). Ketogenic Diet Intervention for Obesity Weight-Loss- A Narrative Review, Challenges, and Open Questions. Curr. Nutr. Rep..

[120] Hendrie G.A., Baird D.L., James-Martin G., Brindal E., Brooker P.G. (2025). Correction: Weight Loss Patterns and Outcomes Over 12 Months on a Commercial Weight Management Program (CSIRO Total Wellbeing Diet Online): Large-Community Cohort Evaluation Study. J. Med. Internet Res..

